# Decreased Thioredoxin-1 and Increased HSP90 Expression in Skeletal Muscle in Subjects with Type 2 Diabetes or Impaired Glucose Tolerance

**DOI:** 10.1155/2014/386351

**Published:** 2014-02-04

**Authors:** M. Venojärvi, A. Korkmaz, S. Aunola, K. Hällsten, K. Virtanen, J. Marniemi, J.-P. Halonen, O. Hänninen, P. Nuutila, M. Atalay

**Affiliations:** ^1^Institute of Biomedicine, Exercise Medicine, University of Eastern Finland, P.O. Box 1627, 70211 Kuopio, Finland; ^2^Institute of Biomedicine, Physiology, University of Eastern Finland, P.O. Box 1627, 70211 Kuopio, Finland; ^3^Department of Health, Functional Capacity and Welfare, Functional Capacity Unit, National Institute for Health and Welfare, 20720 Turku, Finland; ^4^PET Centre, University of Turku, 20520 Turku, Finland; ^5^Department of Chronic Disease Prevention, Population Studies Unit, National Institute for Health and Welfare, 20520 Turku, Finland; ^6^Social Insurance Institution, Research Department, 20720 Turku, Finland

## Abstract

In diabetes, the endogenous defence systems are overwhelmed, causing various types of stress in tissues. In this study, newly diagnosed or diet-treated type 2 diabetics (T2D) (*n* = 10) were compared with subjects with impaired glucose tolerance (IGT) (*n* = 8). In both groups, at resting conditions, blood samples were drawn for assessing metabolic indices and skeletal muscle samples (*m. vastus lateralis*) were taken for the measurements of cellular defence markers: thioredoxin-1 (TRX-1) and stress proteins HSP72, HSP90. The protein level of TRX-1 was 36.1% lower (*P* = 0.031) and HSP90 was 380% higher (*P* < 0.001) in the T2D than in the IGT subjects, with no significant changes in HSP72. However, after the adjustment of both analyses with HOMA-IR only HSP90 difference remained significant. In conclusion, level of TRX-1 in skeletal muscle tissue was lower while that of HSP90 was higher in T2D than in IGT subjects. This may impair antioxidant defence and lead to disruptions of protein homoeostasis and redox regulation of cellular defences. Because HSP90 may be involved in sustaining functional insulin signalling pathway in type 2 diabetic muscles and higher HSP90 levels can be a consequence of type 2 diabetes, our results are potentially important for the diabetes research.

## 1. Introduction

Increased oxidative stress is defined as excessive production of reactive oxygen species (ROS) overwhelming the endogenous antioxidant production in tissues and possibly impairing cellular functions. Hyperglycaemia stimulates the ROS production; both lipid peroxidation and protein oxidation have been shown to be increased in type 1 and type 2 diabetes [1]. At lower concentrations, ROS also serve as secondary messengers regulating cellular functions and adaptations in skeletal muscle tissue [2]. The redox states of thiol systems are controlled by thioredoxin (TRX), glutathione (GSH) and cysteine (Cys); an imbalance between ROS and antioxidant defence appears as aberrant cell signalling and dysfunctional redox control [3].

ROS also induce heat shock protein (HSP) expression and upregulate the HSP defence mechanism, which principally restores protein homeostasis, promotes cell survival, and also provides an additional protection system against overwhelming ROS [4]. HSP90 regulates the heat-shock response and, if this has been inhibited, HSP70 will be upregulated [5]. TRX, a major cellular protein disulfide reductase, modulates protein structure and aggregation by cross-linking proteins with disulfides and reduction of protein cysteine residues. Therefore, in addition to the crucial functions of TRX in tissues in supporting the large network of antioxidant defence, TRX has also crucial role in regulating various protein functions, including enzyme activity, cell growth, proliferation, and ultimately redox-sensitive signal transduction [6, 7]. TRX protects cells from apoptosis [8] and controls many inflammatory genes through redox regulation of transcription factors [9].

Impaired HSP and TRX-1 responses exert a negative impact on antioxidant defence and tissue protection in diabetic patients and experimental diabetes [4, 10]. An early study showed that serum TRX levels were higher in type 2 diabetics (T2D) compared to controls [11]. It has been shown that hyperglycaemia, which was induced by oral glucose loading impaired both serum TRX levels and insulinogenic index, is an indicator of pancreatic *β*-cell function [12]. In contrast, in a pooled group of IGT and T2D patients glucose intolerance was independently associated with the high plasma levels of TRX [13]. Therefore, limited literature information on the association of TRX with glucose homeostasis showed controversy and was restricted with plasma and serum levels, while, to our knowledge, TRX responses to impaired glucose homeostasis in skeletal muscle, the main side for insulin action, have not been studied yet.

Previous data on the effect of type 2 diabetes or IGT on HSP levels were partly limited on mRNA results and conflicting with protein levels: decreased at mRNA levels [14, 15], but increased at protein levels in type 2 diabetics [16]. Furthermore, HSP protein levels were lower in skeletal muscle of insulin resistant, obese humans compared to healthy controls [17]. As far as we are aware, there is no study comparing the levels of HSP and TRX-1 protein levels in skeletal muscle between impaired glucose tolerance (IGT) and T2D subjects. For this purpose in the current study, we evaluated the skeletal muscle protein expressions of HSP72, HSP90, and TRX-1 between subjects with a newly diagnosed or diet-treated type 2 diabetes (T2D) and prediabetic subjects with IGT.

## 2. Material and Methods

### 2.1. Study Subjects

Newly diagnosed or diet-treated T2D subjects (*n* = 10) from a study of Hällsten et al. [18] were compared with a group of IGT subjects (*n* = 8) from a substudy of Finnish Diabetes Prevention Study (DPS) [19–21]. Both studies have been described in detail elsewhere [18, 21]. The study protocol [18] was briefly as follows. A total of 45 patients having T2D, as defined by the criteria of the World Health Organization [22], but no diabetic complications were assigned to the protocol. Patients with a cardiovascular disease, blood pressure > 160/100 mmHg, previous or present abnormal hepatic or renal function, antidiabetic medication, anaemia, or oral corticosteroid treatment were excluded. Age, BMI, and gender matched IGT subjects (*n* = 8) from a study of Venojärvi et al. [21] were included in the present study. There was no difference in maximal oxygen uptake (VO_2max⁡_, mL/kg^−1^/min^−1^) between the groups. The test was performed as previously described [21]. The characteristics of the subjects are shown in Table 1. The Ethical Committee of the Hospital District of South-West Finland, Turku, Finland, and the Ethical Committee of the Rehabilitation Research Centre of the Social Insurance Institution of Finland approved the protocol of this substudy. All subjects gave their written informed consent to participate.

### 2.2. Blood and Muscle Tissue Samples

Blood samples for assessing the metabolic indices were drawn and skeletal muscle samples (*m. vastus lateralis)* were taken for determining the profile of myosin heavy chains (MHC), TRX and HSPs. Plasma glucose was assayed enzymatically with hexokinase (Olympus System Reagent, Hamburg, Germany) and serum insulin was analyzed with a radioimmunoassay (Pharmacia, Uppsala, Sweden). Insulin resistance was determined by using the Homeostasis Model Assessment for Insulin Resistance (HOMA-IR) and *β*-cell function (HOMA-B), as described by Matthews et al. [23]. Haemoglobin A1c (HbA_1c_) concentration was assayed by using latex immunoagglutination inhibition methodology (DCA, 2000 Reagent Kit, Bayer Corporation, Elkhart, IN, USA). Muscle biopsies were taken in resting conditions (no heavy exercising during the preceding 2 days) from the *vastus lateralis* muscle under local anaesthesia (lidocaine 10 mg/mL). From each subject a percutaneous needle biopsy was performed using the conchotome technique [24] and biopsy materials were further divided into three equal portions. Muscle samples were immediately frozen in liquid nitrogen and stored at −70°C until analyzed. Samples for biochemical analyses were melted in ice-bath, weighed, and homogenized in 1 : 10 (w/v) of 1 M Tris buffer pH adjusted to 7.5, containing 5 *μ*mol/L dithiothreitol and 5 *μ*mol/L phenylmethylsulphoxide, using a manually operated all-glass small Potter-Elvehjem homogenizer. The muscle homogenates were centrifuged at 3000 g for 15 min at 4°C to obtain clear supernatants and stored in multiple aliquots to avoid repetitive thawing and freezing. We, however, did not observe any significant effect of repeated freezing and thawing on any of the Western blot signals.

### 2.3. Determination of Myosin Heavy Chain Profile

The MHC profile consisting of the isoform composition of MHC I, MHC IIa, and MHC IIx was determined from muscle homogenate by using the SDS-PAGE gel electrophoresis and Bio-Rad Protean II Xi vertical slab gel system [25].

### 2.4. Western Blot Assays

Skeletal muscle sample homogenates were stored at –70°C until analyzed. Western blot assays were performed as described previously [4, 21]. Briefly, one-dimensional sodium dodecyl sulfate polyacrylamide (10%) gel electrophoresis was done to separate proteins according to their molecular weight. The blots were incubated overnight at +4°C with the following antibodies: anti-HSP72, anti-HSP-90 (Stressgen, Victoria, BC, Canada), and anti-Thioredoxin (IMCO Corp. AB, Stockholm, Sweden). Horseradish peroxidase-conjugated immunoglobulins were used as secondary antibodies. Antibody binding was viewed by using an enhanced chemiluminescence method (NEN Life Science Products, Boston, MA, USA) and quantified by using the image analysis software (NIHImage, MD, USA). The results were normalized according to beta-actin (LabVision/NeoMarkers, Fremont, CA, USA) values. Coefficient of variation for intra-assay replicate samples was less than 7.3% and for interassay samples less than 11.0% for beta-actin signal in western blot analyses. A control sample was used in all the Westernblot runs. The protein concentration was measured in duplicate from the homogenates using BCA method (Pierce, Rockford, IL, USA), and equal amounts of the protein extracts (20 *μ*g) were loaded in the gels. For BCA assay coefficient of intra- and interassay variations was less than 2.10% and 7.94%, respectively (with mean CV% values of 1.36% and 4.34%, resp.). Equal loading and transfer of the western blot samples were further verified and compared with beta-actin by reversible total protein staining of the nitrocellulose membrane with Ponceau-S reversible membrane staining. Unless otherwise stated, all chemicals and reagents were obtained from Sigma-Aldrich (St. Louis, MO) and were of analytic grade or the highest grade available.

### 2.5. Statistics

Data are reported as means ± standard errors (SE). The Kruskal-Wallis test was used to assess differences between the groups. In addition, we applied analysis of covariance (ANCOVA) that was adjusted for HOMA-IR values to compare the difference in TRX-1 and HSP90 expression between IGT and T2D subjects. Pearson's correlation coefficients were used to express the associations between the variables.

## 3. Results

The T2D subjects had significantly higher fasting glucose (*P* = 0.004) and HbA_1c_ (*P* = 0.006) concentrations than the IGT subjects (Table 1). In contrast, the IGT subjects showed significantly higher fasting insulin (*P* = 0.009) and HOMA-IR (*P* = 0.020) values than the T2D subjects (Table 1). In the *vastus lateralis *muscle, the proportion of MHC I isoforms was higher (*P* = 0.001) and the proportion of MHC IIx isoforms was lower (*P* = 0.009) in the T2D than in the IGT subjects (Table 1).

**Figure 1:**
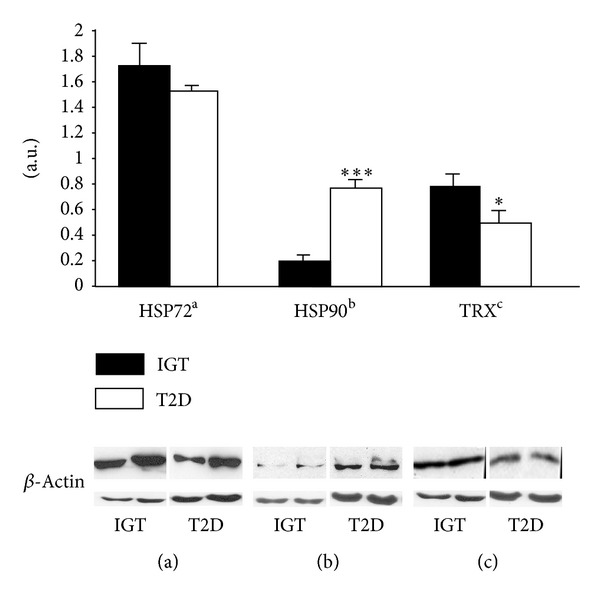
Protein expressions of HSP72, HSP90, and TRX in the *vastus lateralis* muscle of the impaired glucose tolerance (IGT) and type 2 diabetic (T2D) subjects. Data are given as means ± SE. *P < 0.05, ***P < 0.001 between groups. Representative Western blots, using anti-HSP72, anti-HSP90, anti-TRX, and beta-actin antibodies, of whole tissue homogenates from skeletal muscle biopsies of two IGT and T2D subjects. (a.u.) arbitrary units.

The expression of TRX was 36.1% lower (*P* = 0.031) and the expression of HSP90 was 380% higher (*P* < 0.001) in the T2D than in the IGT subjects (Figure 1). After adjusting the TRX and HSP90 expressions with HOMA-IR values, statistically significant differences were found between T2D and IGT subjects in HSP90 (*P* = 0.001). The expression of HSP72 was 10.9% higher in the IGT than in the T2D subjects, but the difference was not statistically significant (*P* = 0.170; Figure 1). The expression of TRX and HSP90 correlated positively in the IGT (*r* = 0.788; *P* = 0.020) but not in the T2D subjects (*r* = −0.193; *P* = 0.593). After adjustment with HOMA-IR, a positive correlation remained between the expressions of TRX and HSP90 in the IGT group (*r* = 0.777, *P* = 0.45). No such correlation was found in the T2D subjects with or without adjustments (*r* = −0.221, *P* = 0.568).

## 4. Discussion

The protein expression of thioredoxin-1 (TRX-1) in the *vastus lateralis *muscle was significantly lower in the type 2 diabetes (T2D) patients than in the IGT subjects. However, after adjustment with insulin resistance index (HOMA-IR), fasting glucose, or insulin levels (data not shown), the significant difference in TRX-1 values between groups disappeared. These observations may be interpreted as that both insulin resistance and hyperglycaemia increase oxidative stress and may be one reason behind the lower TRX-1 expression in the T2D subjects. Previous studies have shown that obese or type 2 diabetic subjects have a higher proportion of type IIb fibers (cf. MHC IIx) in skeletal muscle tissue [26, 27], and, for the insulin sensitivity, the muscle fibers follow the order type I > type IIa > type IIb [28, 29]. However, in our study IGT subjects had a similar MHC profile as in previous reports on obese or T2D subjects. Nevertheless HOMA-IR could be altered and significantly be higher in IGT than T2D subjects. Furthermore, while making a direct comparison of HOMA-IR between IGT and T2D subjects, it has to be kept in mind that T2D subjects are less insulin resistant but their insulin secretion is decreased compared to IGT subjects. In addition, HOMA-IR reflects better liver and whole body insulin resistance than skeletal muscle insulin resistance. A recent study demonstrated that compared to the age-matched healthy controls plasma and lymphocyte levels of TRX-1 were lower in T2D patients with nephropathy complication [16]. In previous studies serum, plasma, and lymphocyte levels of TRX-1 were significantly higher in T2D patients than in healthy controls [11, 16]. However, TRX did not correlate with fasting blood sugar or haemoglobin A1c, although a significant correlation was found between TRX and fasting immunoreactive insulin in the diabetic patients treated with diet/exercise or oral hypoglycaemic agents [11]. In another study a significant correlation has been shown between decreasing serum TRX levels and impaired insulinogenic index, an indicator of pancreatic *β*-cell function in response to hyperglycaemia which was induced by oral glucose loading, while such relationship between TRX levels and insulinogenic index has not been observed at baseline [12]. In contrast to these reports Miyamoto et al. [13] showed that in IGT and T2D patients high plasma concentration of TRX was an independent determinant of glucose intolerance and positively correlated with glycosylated haemoglobin, while no significant difference between IGT and T2D patients in plasma TRX-1 levels was observed. Most of these reports except that of Miyamoto et al. [13] support our results that T2D may lead to an imbalance of antioxidant defence and redox regulation through impaired TRX regulation. In addition, we found a positive correlation between TRX-1 and HSP90 levels in the IGT subject. In the compensated phase of increased oxidative stress (as rather in IGT than T2D), high ROS levels may stimulate synthesis of endogenous antioxidants including TRX and, simultaneously, as a metabolic stress factor, oxidative stress may also induce HSP expression. In T2D further downregulation of antioxidant protection and uncontrolled oxidative stress may cause a more oxidized thiol redox ratio; oxidation of TRX and GSH eventually results in a net loss of intracellular thiol pools which has also been described in other studies [3].

On the other hand, controversy in the literature on TRX responses to impaired glucose homeostasis and lack of difference between IGT and T2D patients could be explained by the fact that in all these studies peripheral levels of TRX-1 have been measured, which may not reflect precisely TRX response in skeletal muscle; the main side for insulin action. Therefore our results may suggest that impaired skeletal muscle TRX-1 response can be a better parameter or even a determinant of the insulin resistance through its function in the network of cytoprotection.

In the present study, the basal expression of HSP72 protein did not differ significantly between the T2D and the IGT subjects. The only study comparing HSP expression between IGT and T2D showed that identical twins with IGT did not have any statistically significant difference in skeletal muscle HSP72 mRNA levels compared with their diabetic cotwins, where HSP72 protein levels were not studied [14]. Nevertheless there is a wide variation in HSP72 responses to T2D and IGT in the literature, where tissue specific response could also be one of the reasons. HSP72 protein levels were reported to be increased in T2D patients in serum [30] and in lymphocytes [16] but HSP72 mRNA was lower in skeletal muscle [15]. Furthermore, HSP72 protein expression was lower in the skeletal muscle of insulin resistant obese humans compared to healthy controls [17]. On the other hand, although HSP70 levels were significantly higher in the patients with T2D for more than 5 years than in newly diagnosed patients, HSP70 was inversely correlated with fasting blood sugar in the patients with diabetes for more than 5 years [30]. These results suggest that although higher HSP levels can be a consequent and a marker of sustained metabolic stress, lower HSP levels may contribute to disrupted glucose homeostasis.

Regarding the association of TRX with HSPs, Calabrese et al. [16] have recently shown that plasma and lymphocyte levels of TRX-1 were lower and lymphocyte inducible HSP70 levels were higher in T2D patients compared to the age-matched healthy controls. In contrast, in our study, we observed a significant decrease of TRX-1 parallel with a nonsignificant (10.9%) decrease in HSP72 levels in skeletal muscle of T2D patients than IGT subjects. Besides the characteristics of different tissues and methodological aspects, the variations in the results can also be explained by the rather large difference in the muscle-fibre composition: myosin heavy chain (MHC) profile between the T2D and the IGT subjects. Thus, it can be assumed that the expression of HSP72 is dependent on the type of muscle MHC profile [31] thereby reflecting the state of insulin resistance. This assumption is supported by the findings of Locke et al. [32], showing that the expression of HSP72 in the basal state is higher in type I fibres than type II fibres in rat skeletal muscle. Similarly heat shock protein (HSP) 72 expression was higher in soleus muscle, composed of type I fibres, and compared with epitrochlearis muscle of type II fibres in Fiscer 344 rats [33]. In addition, training status, physical activity levels, thermal exposure history, energy availability, sex, and age are possible factors that affect the baseline levels of HSP [34]. The aerobic performance capacity of IGT and T2D subjects was similar as their age and BMI. It would strengthen our results significantly if we could have additional information on the training status, thermal exposure history, and energy availability of both study groups. In addition, lack of comparison to healthy normoglycemic subjects is an important weakness of this study, and also a more direct comparison could be possible if the subjects would have similar MCH profile in IGT and T2D groups. In order to confirm the role of the TRX system in the pathogenesis of diabetes, future studies should be designed with larger populations, comparing both thioredoxin related proteins and HSP proteins in different states and phases of impaired glucose regulation in animals as well as in humans.

The HSP90 expression was markedly higher in the *vastus lateralis *muscle in the T2D than in the IGT subjects. HSP90 is expressed in the cytosol, nucleus, and endoplasmic reticulum [35] and plays several physiological roles, such as mediating the stability of protein kinase B (PKB/AKT) and maturation of tyrosine kinase receptors [35–37]. Højlund et al. [38] reported increased HSP90 levels in the skeletal muscle of diabetic subjects, but they did not find any correlation between fasting plasma glucose and HSP90 levels. This may be due to the fact that HSP90 contributes to maintain the activity of protein kinase B, which participates in the regulation of both insulin-stimulated glucose transport and glycogen synthesis [39]. It seems that the expression of HSP90 is markedly upregulated in human skeletal muscle in T2D or corresponding conditions. In future studies, it would be interesting to investigate the causes and consequences of increased HSP levels in diabetes and, more specifically, its relationship to impaired glucose regulation and protein kinase B signalling in animals and humans.

To summarize, in the basal state of skeletal muscle tissue, the protein expression of TRX-1 was significantly lower while that of HSP90 was significantly higher in the T2D patients than in the IGT subjects. Decreased skeletal muscle TRX levels in T2D may lead to an imbalance in the antioxidant defence mechanisms and weaken the HSP protection, which may be associated with the progression of IGT to T2D. Because HSP90 may be involved in the insulin signalling pathway in T2D muscles or can be a consequence of type 2 diabetes and may be sign of oxidative stress which is induced by chronic hyperglycaemia, our results may potentially be important for diabetes research.

## Figures and Tables

**Table 1:** Characteristics of the subjects, myosin heavy chain profiles, and variables of glucose metabolism.

Characteristics	IGT	T2D	*P *value
*n* = (female/male)	8 (4/4)	10 (4/6)	
Age, year	61.4 ± 2.8	62.5 ± 1.6	0.240
BMI	29.7 ± 0.9	29.0 ± 0.5	0.366
VO_2max⁡_, mL/kg^−1^/min^−1^	26.1 ± 2.8	26.5 ± 1.9	0.907
Myosin heavy chain profile			
MHC I, %	37.3 ± 2.0	58.1 ± 5.0	0.001
MHC IIa, %	42.9 ± 2.8	34.8 ± 4.7	0.081
MHC IIx, %	19.8 ± 2.6	7.0 ± 2.1	0.009
Blood chemistry			
Fp-glucose, mmol/L	5.6 ± 0.2	7.1 ± 0.3	0.004
S-insulin, *μ*U/mL	15.4 ± 3.3	8.4 ± 0.5	0.009
HbA_1c_, %*	5.7 ± 0.2	6.4 ± 0.1	0.006
HOMA-IR	5.0 ± 0.9	2.7 ± 0.2	0.020
HOMA-beta %	50.2 ± 5.4	20.3 ± 1.4	0.001

Data are given as means ± SE.

*HbA_1c_ values (in mmol) are 39 and 46 mmol/mol in the IGT and T2D groups respectively (IFCC-HbA_1c_).
